# Circulating Tumor Cells in Gastrointestinal Malignancies: Current Techniques and Clinical Implications

**DOI:** 10.1155/2010/392652

**Published:** 2009-11-05

**Authors:** Georg Lurje, Marc Schiesser, Andreas Claudius, Paul Magnus Schneider

**Affiliations:** ^1^Department of Visceral- and Transplantation Surgery, Department of Surgery, University Hospital Zurich, 8091 Zurich, Switzerland; ^2^Department of Medicine (Cancer Research), West German Cancer Center, University Hospital Essen, 45122 Essen, Germany

## Abstract

Since their introduction more than 50 years by Engell, circulating tumor cells (CTCs) have been evaluated in cancer patients and their detection has been correlated with clinical outcome, in esophageal, gastric, and colorectal cancer. With the availability of refined technologies, the identification of CTCs from peripheral blood is emerging as a useful tool for the detection of malignancy, monitoring disease progression, and measuring response to therapy. However, increasing evidence suggests a variety of factors to be responsible for disease progression. The analysis of a single CTC marker is therefore unlikely to accurately predict progression of disease with sufficient resolution and reproducibility. Here we discuss the current concept of CTCs, summarize the available techniques for their detection and characterization, and aim to provide a comprehensive update on the clinical implications of CTCs in gastrointestinal (GI) malignancies.

## 1. Introduction

Circulating tumor cells (CTCs), also known as the “leukemic phase” of solid tumors, comprise the hematogenous route of metastasis and have been associated with clinical outcome in various malignancies, including breast, esophageal, gastric, and colorectal cancer.

Tumor cells, which detach from the primary tumor into the bloodstream and travel via circulation to distant sites, where they potentially develop into secondary tumors, can cause metastatic lesions, which are a major source of mortality in patients with cancer. The detection of malignant cells in blood has been established since years [1], and recent studies have demonstrated the malignant nature of CTCs [2, 3]. This includes the following steps; tumor growth and neovascularization (tumor-associated angiogenesis), tumor invasion and epithelial to mesenchymal transition (EMT), intravasation, dissemination, arrest in organs, extravasation, proliferation, and formation of distant metastases. Tumors of epithelial or hematopoietic origin show a different tendency to metastasize; where some tumors show no or delayed metastatic spread, others show metastases at the time of diagnosis.

With the availability of refined technologies, the identification of CTCs from peripheral blood is emerging as a useful tool in the detection of particular malignancies, monitoring disease progression, and measuring response to therapy. This review will discuss the current value of CTCs, summarize the currently available techniques for their detection and characterization, and should provide a comprehensive update on their clinical implications in gastrointestinal (GI) malignancies.

## 2. The Concept of Circulating Tumor Cells

Even though most solid tumor patients undergo multimodality therapies (e.g., various combinations of surgery, chemotherapy, and radiation therapy), dissemination of malignant tumor cells, and metastatic disease continues to be the major cause of cancer related death. As a primary tumor grows, neovascularization and sustained tumor angiogenesis, the formation of new blood vessels from endothelial precursors, are prerequisites for the growth and progression of solid malignancies [4]. Initially, tumors grow as avascular masses, which can proliferate on pre-existent vasculature within the microenvironment. When a tumor grows beyond a certain size of approximately 2-3 mm, the tumor requires its own new and dedicated vasculature [5]. The so-called “angiogenic switch,” the induction of tumor vasculature or switch to an angiogenic phenotype, is considered a hallmark of the malignant process and is required for tumor propagation and progression [6–8]. Tumor cells may invade the neighbouring normal blood vessels that were already in place at the primary tumor site, or they may use the new-formed capillaries that grow intrinsic to the tumor as a result of tumor-associated angiogenesis. In either case, there is a tissue und tumor specific induction of epithelial to mesenchymal transition (EMT), a change in the expression of cell adhesion molecules (e.g., integrins, laminins) and activation of proteases pathways (e.g., matrix metalloproeteinases) that eventually allow tumor cells to enter the host circulation [9].

Since Stephen Paget's historic discovery of the “seed and soil” concept, namely, CTCs tend to metastasize to certain organ sites, which are selective for cells derived from specific tumors [10], research on a global scale has attempted to unravel the underlying molecular mechanisms that bring “seed and soil” together to promote metastases [11, 12]. A current definition of the “seed and soil” hypothesis comprises three principles; at first, neoplasms contain genetically diverse tumor cell subpopulations, each with different metastatic potential. Secondly, metastases will be formed by those cells, which will succeed in completing all steps in the metastatic process and thirdly, the specific choice of “soil” is mostly attributed to interactions between the tumor cell and the organ microenvironment. These interactions include tumor cell specific recognition of endothelial cell antigens and response to local growth factors [11].

### 2.1. Immune System and CTC Interaction

Since CTCs and their primary tumor are generally considered as foreign tissue for the patients immune system, they have the ability to form clusters of microtumors, thus escaping the hosts immune surveillance and gain a selective growth advantage over local cells at the distant site [9]. Indeed, aggregation of 5–10 occult CTCs has been shown to escape the hosts immune system, potentially promoting the recruitment of proangiogenic (growth) factors from the local microenvironment and expression of new cell surface markers [13]. In fact, some CTCs show heterogenic expression patterns of cell surface markers in comparison to their primary tumors. As such, CTCs may affect the interaction with the patients immune defense, making it somewhat difficult to predict the “biologic fate” of CTC-derived microtumors [13].

### 2.2. Local Invasion, Tumor-Dissemination, Extravasation/ Intravasation, and Implantation

Many aspects of disseminated tumor cells remain to be determined. It is well recognized though that metastatic disease is predominantly driven by the tumors molecular characteristics (seed) and hosts' microenvironment (soil) [10, 11]. The unregulated growth of tumors is attributed to the serial acquisition of genetic events, which are characterized at least in part by enhanced cell proliferation, silencing of genes involved in the inhibition of cell differentiation and circumventing genes involved in apoptosis [14]. Differentiated cells in adult bowel epithelium demonstrate only a short life-span with a complete self-renewal time of approximately 2–7 days [15]. Due to their longevity and self-renewing properties “cancer stem cells,” a small subgroup of cells in malignant tumors, have a greater propensity to accumulate these carcinogenic mutations [16, 17]. They are therefore considered responsible for tumor growth and progression [18–20]. This enables a complex crosstalk between cells from the adjacent structures, including the stroma, and cancer cells. Even before cancer cells penetrate the basal membrane and become invasive, they succeed to stimulate neovascularization on the stromal side of the membrane, apparently through dispatching angiogenic factors, such as VEGF and EGF [5]. In addition, tumor necrosis leads to the recruitment of inflammatory cells that may enhance the local production of chemokines (IL-8, IL1B) and growth factors by adjacent stroma cells [7, 21]. As a result, matrix metalloproteinases (MMPs) are produced by inflammatory cells within the adjacent stroma and are capable of degrading the extracellular matrix (ECM), further facilitating local invasion and phenotypic alterations of the tumor, ultimately leading to tumor cell dissemination and metastatic disease [22].

Tumor cells that have passed the basal membrane are highly mobile and quickly penetrate the vascular endotheliums or lymphatic vessels (intravasation) where they circulate as CTCs and may extravasate at distant organ sites. These circulating tumor cells show a geno- and phenotypic variability. In fact, there is accumulating evidence that some of them are apoptotic, while others express markers of “pluripotent progenitor cells” [23, 24]. Once the target organ is reached, CTCs often change to their more epithelial phenotype suppressed during the EMT [25]. In this way the mesenchymal-like CTCs can undergo mesenchymal-epithelial transition (MET) and regain the ability to proliferate [26]. In some cases, tumor cells can also invade as multicellular clusters or microtumors in a process termed “collective migration” [27]. These microtumors are like circulating tumor microemboli (CTM) and are thought to have a high metastatic potential. As such, they may give rise to metastases without extravasation by attaching to the walls of vessels and proliferating within the vasculature and ultimatively induce the rupture of these vessel walls. As such micro- and macrometastasis may derive either from extravasated CTCs or from nonextravasated CTMs after limited proliferation and rupture of vessel walls [28, 29].

## 3. Methodological Considerations for the Detection of CTCs

A variety of methods are currently available for enriching and detecting CTCs from peripheral blood [3, 29]. However, their detection and characterization has been hindered by the usage of nonstandardized, often nonspecific, and\or nonsensitive detection methods leading to discordant study results. In fact, no ideal method is currently available and several limitations apply to each of the following methods described. Some of them however may be overcome if they are used in combination [25, 30–33]. The most common techniques for CTC enrichment and detection are discussed as follows (Table 1).

### 3.1. Density Gradient Separation of Mononucleated Cells

Density gradient separation of mononucleated cells and CTCs from other cells in the blood is performed using commercially available kits such as Lymphoprep (Nycomed, Oslo, Norway), OncoQuick (Greiner, Frickenhausen, Germany), or other similar density gradient liquids. This process generates a layered separation of cell types based on the cellular density. After centrifugation, from bottom to top, the following layers are found: erythrocytes, neutrophils, density gradient, mononuclear cells, and plasma, which is the top layer. Although the addition of a porous barrier (OncoQuick) above the density gradient has prevented its mix with whole blood, there are still limitations with this method. These particularly include migration of CTCs into the plasma fraction and the formation of aggregates at the bottom of the gradient.

### 3.2. Direct Enrichment of Circulating Epithelial Cells by Filtration

Direct enrichment of epithelial cells by filtration is based on the observation, that the vast majority of peripheral blood cells belong to the smallest cells in the human body (8 to 11 um). They can be eliminated by blood filtration through polycarbonate membrane calibrated pores of 8 um [29]. Even though this method involves only one single step, it is not highly effective in the process of enriching CTCs.

### 3.3. Immunomagnetic Cell Enrichment

Immunomagnetic cell enrichment is the most commonly used technique for CTC enrichment. This approach can be performed by itself or integrated with other physical (density gradient, direct filtration) or nonphysical downstream molecular methods such as quantitative real-time reverse transcription polymerase chain reaction (RT-PCR) or fluorescence in situ hybridization (FISH). Several protocols have been developed and are now commercially available. Their yield ranges from 1 × 10^4^- to 2 × 10^5^-fold CTC enrichment and in contrast to RT-PCR usually avoid cell lysis, thus allowing a direct count of CTCs [29]. These systems rely on positive selection of CTCs from samples through their binding of antibodies coupled to magnetic beads that target epithelial- or tumor-specific cell surface antigens, such as cytokeratins (CKs), human epithelial antigens, epithelial cell adhesion molecules, as well as prostate specific antigen (PSA), carcinoembryonic antigen (CEA), human epithelial growth factor receptor-2 (HER-2). Since no available antibody is 100% tumor or tissue specific [45], false-positive (specifity) and false-negative (sensitivity) results continue to impose a significant problem with immuno-magnetic detection techniques. Recently, negative selection of white blood cells by CD45 and CD61 specific antibodies in addition to aforementioned positive selection methods was applied in an attempt to further increase specific enrichment of CTCs from peripheral blood (CellSearch, Veridex LLC, Raritan, NJ, USA) [33, 36, 46]. The use of semiautomated devices for the microscopic screening of large amounts of immunostained slides has already helped to increase speed and reproducibility of immuochemical analyses [47]. Among the commercially available semiautomated approaches, the CellSearch system (Veridex LLC, Raritan, NJ, USA), which has been federal drug and food administration- (FDA-) approved for the use in metastatic breast and colon cancer patients, has gained considerable attention lately, because it allows both automated immunomagnetic epithelial cell adhesion molecule-based (EpCAM) enrichment, as well as cyotokeratin staining of CTC in blood samples. Even though this technique eliminates the majority of leukocytes, the presence of nonepithelial cells and CTCs that do not express epithelial antigens (by EMT) is still considered a major pitfall of this approach. The major advantages of immunomagnetic separation are the direct visualization and quantification of CTCs and the detection of living cells without the need of cell lysis, as it is the case for PCR-based methods.

### 3.4. Reverse-Transcriptase Polymerase Chain Reaction (RT-PCR)

Detection of CTCs from peripheral blood of cancer patients by conventional reverse-transcriptase PCR (RT-PCR) has been reported for several genes, for example, CEA, CK-19, or -20 [48, 49]. Since its introduction almost 20 years ago, RT-PCR has emerged as the most common used technique for the detection of CTCs [25]. The main advantage of this approach is its sensitivity that is considered higher than other currently available protocols. This is particularly true for protocols where mRNA is isolated after enrichment of CTCs from whole blood [31]. As such, RT-PCR is often considered the most sensitive assay to detect tumor-specific molecular markers. In addition, RT-PCR does also offer high specifity, as primers are designed for the particular gene of interest and the whole genomic DNA or RNA can be analyzed in one single reaction. Furthermore, RT-PCR of tumor-specific mRNA is characterized by a higher sensitivity in comparison to protein-based methods and usually ranges within a detection range of 1 to 10 tumor cells among 10^6^-10^7^ blood mononuclear cells [50, 51]. Nevertheless, the high sensitivity of RT-PCR, although important for its clinical use, is problematic when false positive results are encountered, as it is the case with sample contamination (genomic DNA versus cDNA), illegitimate transcription (low-level nonspecific transcription of certain genes), or amplification of pseudogenes [52, 53]. Another limitation is the selection of mRNA markers used, since the ideal marker would likely be highly overexpressed in all tumor cells from a given tumor, but not expressed at all in white blood cells and nonepithelial circulating cells. Indeed there are several studies, which report detection of CK19, CK20, CEA, and PSA in circulating nontumerous (epithelial) cells [38, 51–55]. Therefore, it is extremely important to carefully design primer and probes, choose the “right candidate genes” and ideally combine some of the aforementioned physical enrichment techniques with molecular based methods such as RT-PCR or FISH [33].

### 3.5. Microchip Technology (CTC-Chip)

Newer methods for enrichment have recently been reported that utilize microfluidic platforms (the “CTC-chip”) capable of efficient and selective separation of viable CTCs from peripheral whole blood samples [56]. Nagrath et al. described the “CTC-chip” that separates CTCs in whole blood using EpCAM coated microposts and controlled laminar flow conditions to ensure optimal interaction between the cells and the microposts. Since the CTC-chip can process whole blood directly, the authors claim that a level of purity, that is up to 100-fold higher than what other technologies offer can be achieved [56]. Nevertheless, further validation within prospective clinical trials is warranted.

## 4. Clinical Implications of CTCs in Gastrointestinal Malignancies

The presence of CTCs in patients with metastatic carcinoma is generally associated with poor clinical outcome [9]. Initially, this has been shown for malignancies outside the GI tract, such as breast cancer. Cristofanilli et al. demonstrated that patients with metastatic breast cancer could be segregated into favorable and nonfavorable groups (with reference to PFS—progression free survival and OS—overall survival) based on their CTC count. This was assessed by an immuno-magnetic separation technique (CellSearch, Veridex LLC, Raritan, NJ) that led to FDA approval for the use in metastatic breast cancer in 2005 [36].

In a prospective multicenter study by Cristofanilli et al. 177 patients with measurable metastatic breast cancer (as assessed according to the criteria of the World Health Organization) were tested for blood levels of CTCs, both before the patients were to start a new line of treatment and at the first follow-up visit. Outcomes were assessed according to levels of circulating tumor cells at baseline, that is, before the patients started a new treatment for metastatic disease. Patients with levels of CTCs equal to or higher than 5 per 7.5 mL of whole blood, as compared with the group with fewer than 5 circulating tumor cells per 7.5 mL, had a shorter progression-free survival (2.7 months versus 7.0 months, *P* < .001) and shorter overall survival (10.1 months versus > 18 months, *P* < .001). This difference between the groups persisted (progression-free survival, 2.1 months versus 7.0 months; *P* < .001; overall survival, 8.2 months versus > 18 months; *P* < .001) at the time of the first follow-up visit, suggesting that the reduced proportion of patients (from 49 percent to 30 percent) in the group with an unfavorable prognosis showed a benefit from therapy. The authors concluded that the number of CTC's before treatment is an independent predictor of PFS and OS in patients with metastatic breast cancer [36].

Compared to breast cancer, there are fewer studies available in GI-cancer and the results are less consistent (Table 2). However, given the recent focus on how k-ras mutations affect clinical outcome in metastatic colorectal cancer and anti-EGFR therapy with cetuximab [39–41, 57], evaluation of patients for mutations in k-ras is rapidly becoming part of routine practice in clinical oncology and had so far mostly relied on formalin-fixed paraffin-embedded (FFPE) tumor tissue. Testing of circulating tumor DNA in peripheral blood to screen for mutations resident in the parent tumor (blood biopsy) is unencumbered by many of the factors that limit testing of FFPE-derived specimens. As such, blood is easily accessible, not prone to selection bias, and provides a continuous source of DNA. Accordingly, tests for circulating tumor DNA are able to screen for mutations, such as for mutations of the k-ras oncogene and are present at the time of treatment unlike tests that rely on archived tissue samples that were acquired previously [42, 58].

### 4.1. CTCs in Esophageal Cancer

Even though not many studies have been conducted and results have been inconsistent, the majority of studies that have explored CTCs as a molecular marker for clinical outcome have demonstrated their clinical utility [31, 43, 59, 60]. In fact, a recent study by Liu et al. aimed at establishing a quantitative system for evaluating the role of CTCs in peripheral blood from patients who underwent surgical resection for the treatment of esophageal cancer [43]. One hundred fifty-five peripheral blood samples from 53 esophageal cancer patients were collected before surgery (B − 1), immediately after surgery (B0), and on the 3rd day postoperatively (B + 3). A direct quantitative real time RT-PCR method based on carcinoembryonic antigen (CEA) mRNA gene expression was designed for the detection of CTCs. The authors showed a significant difference between B − 1 and B0 (*P* = .0001) and between B − 1 and B + 3 (*P* = .0209). Fifty percent of patients with *R* > 0.4 (*R* = CTC ratio of B + 3 over B0) showed tumor recurrence within 1 year after surgery, whereas the probability was only 14.3% for patients with *R* < 0.4 (*P* = .043) [43]. These findings are consistent with a recent report for members of our group exploring CTCs from peripheral blood as assessed by a direct quantitative RT-PCR method, using CTC survivin-mRNA expression as a surrogate for CTC count in peripheral blood [31, 60]. In their preliminary findings, the authors could show that direct quantitative real-time RT-PCR analysis of survivin mRNA expression in peripheral blood of patients with gastrointestinal cancers is technically feasible and that survivin mRNA levels fall signifcantly, following complete surgical resection (R0-resection according to UICC) and may be a promising molecular marker for the completeness of surgical resection (molecular R0 marker) [31]. A follow-up study by the same group analyzed peripheral blood samples from 62 esophageal cancer patients, who were scheduled for surgical resection. 25 (40.3%) patients had squamous cell carcinomas and 37 (59.7%) adenocarcinomas. In 24 (38.7%) patients neoadjuvant chemoradiation was performed for locally advanced disease. Whole blood was drawn one day before the operation and 10 days post R0-resection in all patients. Postoperative survivin levels were significantly lower than preoperative levels in 41.2% of resected patients. In patients receiving neoadjuvant chemoradiation a decrease of postoperative survivin mRNA expression was detected in 83.3% of patients with adenocarcinomas compared to 50% with squamous cell carcinoma (Mann-Whitney test: *P* < .04 ), suggesting that survivin levels were particularly decreased in adenocarcinomas following neoadjuvant chemoradiation and surgical resection. Other studies by the same group revealed that esophageal cancer patients who showed minor histopathological response to neoadjuvant radiochemotherapy demonstrated significant higher CTC levels as assessed by ERCC1 mRNA-expression levels in their blood [44]. Furthermore, similar results could be shown for death-associated protein kinase (DAPK) and adenomatous polyposis coli gene (APC) methylation status as assessed by methylation-specific real-time RT-PCR. In fact, 36 out of 59 patients (61.0%) with esophageal cancer had detectable levels of methylated DAPK or APC promoter DNA and preoperative detection was significantly associated with an unfavorable prognosis in the multivariable model [44].

### 4.2. CTCs in Gastric Cancer

Gastric adenocarcinoma is the forth most common type of cancer and the second leading cause of cancer-related death worldwide [61]. Even though combined chemoradiation following complete surgical resection has offered modest improvements in OS and PFS (MAGIC-trial) [62], tumor recurrence after curative resection continues to be a significant problem in the management of patients with localized gastric adenocarcinoma. In an attempt to identify markers for tumor recurrence Miyazono et al. investigated blood samples from 57 patients with gastric cancer for the presence of CTCs [63]. After density gradient separation of CTCs, CEA-specific real-time RT-PCR was performed and correlated with the time course during a surgical procedure and the advent of hepatic tumor recurrence. Interestingly, the authors could show that CEA-mRNA could not be detected in a control group of healthy volunteers and 15 patients with benign disease. In contrast, a total of 21 gastric cancer patients (36.8%) were positive for CTC as detected by CEA-specific RT-PCR and positive rates correlated with depth of tumor invasion. Furthermore, the authors found that gastric cancer patients with high levels of CEA were more likely to develop systemic disease. More recently, Ikeguchi and Kaibara confirmed these findings; even though the authors noted that CTCs may be eliminated very quickly from the hosts circulation and as such may be present only for a very short period of time [64]. Other groups have reported similar findings for relatively heterogenic study populations of patients with gastrointestinal malignancies [65].

### 4.3. CTCs in Colorectal Cancer

CTCs have been studied in colorectal cancer (CRC) [30, 66] and recent studies have shown their clinical utility in patients with metastatic CRC [33, 67]. In a prospective multicenter clinical trial, 430 patients beginning a new first-, second-, or third-line systemic therapy had peripheral blood obtained for enumeration of CTCs (CellSearch, Veridex LLC, Raritan, NJ, USA) at baseline (pretreatment) and subsequent time points. As demonstrated by the authors, CTC count at baseline, and during therapy, was the strongest independent prognostic marker compared with other clinical factors for PFS and OS [33]. As the patient population was heterogeneous in this study, the authors reported on the same study cohort with extended follow-up time to evaluate the prognostic significance of baseline CTC count by line of therapy, type of therapy, and other important clinical characteristics [67]. Interestingly, the results remained remarkably consistent in comparison to the original trial, with a near doubling in PFS and OS for patients with favorable compared to unfavorable baseline CTCs. In addition, elevated baseline CTC count within patient and clinical subgroups was associated with adverse clinical outcome in all subgroups. PFS was also generally inferior in patients with elevated baseline CTCs, even though this finding did not reach statistical significance at the *P* < .05 level. Therefore, the authors state that while an elevated CEA may be a poor prognostic factor for resectable CRC [68], no present data support its prognostic value for metastatic CRC. Thus, the authors suggest that the next large metastatic CRC trial evaluating a new systemic therapy should utilize CTCs as a stratification factor for OS [67].

## 5. Summary and Future Perspectives

Progress in cancer biology has led to an expansion of our understanding of the molecular and cellular mechanisms of cancer development, cancer metastases, and resistance of cancer cells to (radio-) chemotherapy. Despite recent advancements in the detection, surgical resection, and (neo-) adjuvant (radio-) chemotherapy, selection of the most beneficial treatment strategies in gastrointestinal malignancies remains a challenge and is also hindered by the lack of validated predictive and prognostic molecular markers [68]. A multidisciplinary approach, including surgery, tailored (radio-) chemotherapy, alone or in combination will be necessary to improve the outcome for patients with GI cancer. In addition, the high incidence of tumor drug resistance remains a major stumbling block for effective cancer treatment. For the clinician, it is important to distinguish between prognostic and predictive molecular markers. Predictive markers are associated with treatment specific therapy and are mostly evaluated through clinical response, time to progression, or toxicity. In contrast, prognostic markers reflect the nature/aggressiveness of the disease, independently of a specific treatment, and are usually evaluated in terms of OS. In some cases, predictive markers can also carry prognostic weight [69], and both play important roles in the prospective evaluation for a given treatment regimen.

Over the last decades several candidate molecular markers have been discovered. Nevertheless, for almost all of these markers it is necessary to obtain tumor tissue for molecular analysis by at least a biopsy. In contrast, a noninvasive molecular marker, such as CTCs [67], germline polymorphisms [70], or DNA-methylation [71] may offer a quick and noninvasive approach. For instance, monitoring of pre- and postoperative CTC survivin-mRNA levels may provide important clinical utility in predicting the completeness of a surgical resection (molecular R0 marker) [31]. Although tumor markers such as CEA are widely used for the follow-up of patients with GI malignancies, their lack of sensitivity remains unsolved. As such, detection and characterization of CTCs may offer a novel approach, that can be used for the estimation of risk for tumor recurrence and metastatic disease, stratification of patients to chemotherapy, identification of novel therapeutic targets, and monitoring of systemic anticancer therapies. Nevertheless, it is becoming increasingly apparent that disease progression depends on numerous complex pathways, and that the analysis of one single CTC-marker is unlikely to precisely predict progression of disease with sufficient accuracy and reproducibility. In addition, currently available studies are hampered by small sample sizes, heterogeneous patient populations, and most importantly the lack of standardized methodologies for the detection and characterization of CTCs. Ongoing and future clinical trials hold promise for further improvements in optimizing and specifying (radio-) chemotherapy individually, not only prolonging lives but also improving quality of life.

## Figures and Tables

**Table 1:** Common techniques for CTC-enrichment and detection. MACS (Magnetic Activated Cell Separation), ISET (Isolation by Size of Epithelial Tumor Cells), FISH (Fluorescent in Situ Hybridization), RT-PCR (Reverse Transcription Polymerase Chain Reaction).

System	Blood volume per test (mL)	Time for enrichment (minute)	Principle of CTC enrichment and detection	Sensitivity	Reference
OncoQuick	15–35 mL	45 minutes	Cellular density gradient, immunolabeling	1 cell/4.5 uL	[31, 34]
MACS	5–15 mL	120 minutes	Capture by immunobeads, immunolabeling and FISH or RT_PCR	1 cell/0.3 uL	[35]
CellSearch	7,5 mL	40 minutes	Capture by immunobeads (negative selection by CD-45; positive selection by CK-8, -18, -19)	1 cell/0.5 uL	[33, 36]
ISET	10 mL	15 minutes	Direct filtration and immunolabeling	1 cell/mL	[37]

**Table 2:** CTCs studies and clinical outcome in gastrointestinal malignancies.

Patients analyzed	Type of cancer	Method of CTC detection	Results	Reference
53 esophageal cancer patients 22 benign patients who underwent thoracotomy30 healthy volunteers	Esophageal cancer	(1) Density gradient separation (OncoQuick) (2) Real-time RT-PCR (CEA-based)	Patients with high levels of CTCs (CEA) were more likely to show recurrent disease	Liu et al. [43]
62 esophageal cancer patients (25 SCC/37 EA)	Esophageal cancer	(1) Density gradient separation (OncoQuick) (2) Real-time RT-PCR (survivin-based)	Survivn mRNA levels fall after surgical resection	Hoffmann et al. [60]
44 patients with GI malignancy* *-24 esophageal cancer* *-5 gastric cancer* *-8 colorectal cancer* *-3 pancreatic	GI malignancies	(1) Density gradient separation (OncoQuick) (2) Real-time RT-PCR (survivin-based)	Survivn mRNA levels fall after surgical resection	Hoffmann et al. [31]
29 locally advanced esophageal cancer patients (11 SCC/18 EA)	Esophageal cancer	(1) Density gradient separation (OncoQuick) (2) Real-time RT-PCR (ERCC1-based)	ERCC1 mRNA expression associated with response to neoadjuvant radiochemotherapy	Brabender et al. [71]
59 locally advanced esophageal cancer patients (24 SCC/35 EA)	Esophageal cancer	(1) Density gradient separation (2) Methylation specific real-time RT-PCR (DAPK and APC-based)	DAPK and APC methylation associated with unfavourable prognosis	Hoffmann et al. [44]
57 gastric cancer patients 15 patients with benign diseae 15 healthy volunteers	Gastric cancer	(1) Density gradient separation (2) Real-time RT-PCR (CEA-based)	Patients with high levels of CTCs (CEA) were more likely to show recurrent disease	Miyazono et al. [63]
59 gastric cancer patients 15 patients with benign diseae	Gastric cancer	(1) CTC-enrichment was not performed (2) Real-time RT-PCR (CEA-based)	High levels of CEA were associated with lower risk of tumor recurrence	Ikeguchi et al. [64]
430 mCRC patients beginning a new first-, second-, or third-line systemic therapy	Colorectal cancer	Immunomagnetic CTC enumeration (CellSearch, Veridex)	Patients with high CTCs had poor PFS and OS	Cohen et al. [66]
430 mCRC patients beginning a new first-, second-, or third-line systemic therapy (follow-up study)	Colorectal cancer	Immunomagnetic CTC enumeration (CellSearch, Veridex)	Patients with high CTCs had poor PFS and OS (also within subgroups with longer follow up)	Cohen et al. [67]

## References

[1] Engell HC (1955). Cancer cells in the circulating blood; a clinical study on the occurrence of cancer cells in the peripheral blood and in venous blood draining the tumour area at operation. *Ugeskr Laeger*.

[2] Ulmer A, Schmidt-Kittler O, Fischer J (2004). Immunomagnetic enrichment, genomic characterization, and prognostic impact of circulating melanoma cells. *Clinical Cancer Research*.

[3] Fehm T, Sagalowsky A, Clifford E (2002). Cytogenetic evidence that circulating epithelial cells in patients with carcinoma are malignant. *Clinical Cancer Research*.

[4] Folkman J (1971). Tumor angiogenesis: therapeutic implications. *The New England Journal of Medicine*.

[5] Folkman J (1995). Angiogenesis in cancer, vascular, rheumatoid and other disease. *Nature Medicine*.

[6] Lurje G, Zhang W, Lenz H-J (2007). Molecular prognostic markers in locally advanced colon cancer. *Clinical Colorectal Cancer*.

[7] Lurje G, Zhang W, Schultheis AM (2008). Polymorphisms in *VEGF* and *IL-8* predict tumor recurrence in stage III colon cancer. *Annals of Oncology*.

[8] Strieter RM (2005). Masters of angiogenesis. *Nature Medicine*.

[9] Elshimali YI, Grody WW (2006). The clinical significance of circulating tumor cells in the peripheral blood. *Diagnostic Molecular Pathology*.

[10] Paget S (1889). The distribution of secondary growths in cancer of the breast. *The Lancet*.

[11] Fidler IJ (2003). The pathogenesis of cancer metastasis: the ‘seed and soil’ hypothesis revisited. *Nature Reviews Cancer*.

[12] Fidler IJ, Poste G (2008). The “seed and soil” hypothesis revisited. *The Lancet Oncology*.

[13] Mukai M (2005). Occult neoplastic cells and malignant micro-aggregates in lymph node sinuses: review and hypothesis. *Oncology Reports*.

[14] Al-Hajj M, Clarke MF (2004). Self-renewal and solid tumor stem cells. *Oncogene*.

[15] Brittan M, Wright NA (2004). Stem cell in gastrointestinal structure and neoplastic development. *Gut*.

[16] Gudjonsson T, Magnusson MK (2005). Stem cell biology and the cellular pathways of carcinogenesis. *APMIS*.

[17] Pohl A, Lurje G, Kahn M, Lenz H-J (2008). Stem cells in colon cancer. *Clinical Colorectal Cancer*.

[18] Baba M, Natsugoe S, Shimada M (2000). Prospective evaluation of preoperative chemotherapy in resectable squamous cell carcinoma of the thoracic esophagus. *Diseases of the Esophagus*.

[19] Potten CS, Loeffler M (1990). Stem cells: attributes, cycles, spirals, pitfalls and uncertainties. Lessons for and from the crypt. *Development*.

[20] Wong WM, Wright NA (1999). Cell proliferation in gastrointestinal mucosa. *Journal of Clinical Pathology*.

[21] Lurje G, Hendifar AE, Schultheis AM (2009). Polymorphisms in interleukin 1 beta and interleukin 1 receptor antagonist associated with tumor recurrence in stage II colon cancer. *Pharmacogenetics and Genomics*.

[22] Hotz B, Arndt M, Dullat S, Bhargava S, Buhr H-J, Hotz HG (2007). Epithelial to mesenchymal transition: expression of the regulators snail, slug, and twist in pancreatic cancer. *Clinical Cancer Research*.

[23] Pantel K, Brakenhoff RH (2004). Dissecting the metastatic cascade. *Nature Reviews Cancer*.

[24] Schwarzenbach H, Alix-Panabières C, Müller I (2009). Cell-free tumor DNA in blood plasma as a marker for circulating tumor cells in prostate cancer. *Clinical Cancer Research*.

[25] Jacob K, Sollier C, Jabado N (2007). Circulating tumor cells: detection, molecular profiling and future prospects. *Expert Review of Proteomics*.

[26] Kang Y, Massagué J (2004). Epithelial-mesenchymal transitions: twist in development and metastasis. *Cell*.

[27] Wittekind C, Neid M (2005). Cancer invasion and metastasis. *Oncology*.

[28] Al-Mehdi AB, Tozawa K, Fisher AB, Shientag L, Lee A, Muschel RJ (2000). Intravascular origin of metastasis from the proliferation of endothelium-attached tumor cells: a new model for metastasis. *Nature Medicine*.

[29] Paterlini-Brechot P, Benali NL (2007). Circulating tumor cells (CTC) detection: clinical impact and future directions. *Cancer Letters*.

[30] Hoffmann A-C, Brabender J, Metzger R (2009). Dihydropyrimidine dehydrogenase mRNA expression in peripheral blood of rectal cancer patients is significantly associated with residual tumor and distant metastases following resection. *Journal of Surgical Oncology*.

[31] Hoffmann A-C, Warnecke-Eberz U, Luebke T (2007). Survivin mRNA in peripheral blood is frequently detected and significantly decreased following resection of gastrointestinal cancers. *Journal of Surgical Oncology*.

[32] Smerage JB, Hayes DF (2006). The measurement and therapeutic implications of circulating tumour cells in breast cancer. *British Journal of Cancer*.

[33] Cohen SJ, Punt CJA, Iannotti N (2008). Relationship of circulating tumor cells to tumor response, progression-free survival, and overall survival in patients with metastatic colorectal cancer. *Journal of Clinical Oncology*.

[34] Gertler R, Rosenberg R, Fuehrer K, Dahm M, Nekarda H, Siewert JR (2003). Detection of circulating tumor cells in blood using an optimized density gradient centrifugation. *Recent Results in Cancer Research*.

[35] Griwatz C, Brandt B, Assmann G, Zänker KS (1995). An immunological enrichment method for epithelial cells from peripheral blood. *Journal of Immunological Methods*.

[36] Cristofanilli M, Budd GT, Ellis MJ (2004). Circulating tumor cells, disease progression, and survival in metastatic breast cancer. *The New England Journal of Medicine*.

[37] Vona G, Sabile A, Louha M (2000). Isolation by size of epithelial tumor cells: a new method for the immunomorphological and molecular characterization of circulating tumor cells. *American Journal of Pathology*.

[38] Mitsiades CS, Lembessis P, Sourla A, Milathianakis C, Tsintavis A, Koutsilieris M (2004). Molecular staging by RT-PCR analysis for PSA and PSMA in peripheral blood and bone marrow samples is an independent predictor of time to biochemical failure following radical prostatectomy for clinically localized prostate cancer. *Clinical and Experimental Metastasis*.

[39] De Roock W, Piessevaux H, De Schutter J (2008). KRAS wild-type state predicts survival and is associated to early radiological response in metastatic colorectal cancer treated with cetuximab. *Annals of Oncology*.

[40] Di Fiore F, Blanchard F, Charbonnier F (2007). Clinical relevance of *KRAS* mutation detection in metastatic colorectal cancer treated by Cetuximab plus chemotherapy. *British Journal of Cancer*.

[41] Lièvre A, Bachet J-B, Boige V (2008). *KRAS* mutations as an independent prognostic factor in patients with advanced colorectal cancer treated with cetuximab. *Journal of Clinical Oncology*.

[42] Holdhoff M, Schmidt K, Donehower R, Diaz LA (2009). Analysis of circulating tumor DNA to confirm somatic *KRAS* mutations. *Journal of the National Cancer Institute*.

[43] Liu Z, Jiang M, Zhao J, Ju H (2007). Circulating tumor cells in perioperative esophageal cancer patients: quantitative assay system and potential clinical utility. *Clinical Cancer Research*.

[44] Hoffmann A-C, Vallbohmer D, Prenzel K (2009). Methylated DAPK and APC promoter DNA detection in peripheral blood is significantly associated with apparent residual tumor and outcome. *Journal of Cancer Research and Clinical Oncology*.

[45] Goeminne J-C, Guillaume T, Symann M (2000). Pitfalls in the detection of disseminated non-hematological tumor cells. *Annals of Oncology*.

[46] Allard WJ, Matera J, Miller MC (2004). Tumor cells circulate in the peripheral blood of all major carcinomas but not in healthy subjects or patients with nonmalignant diseases. *Clinical Cancer Research*.

[47] Alix-Panabières C, Riethdorf S, Pantel K (2008). Circulating tumor cells and bone marrow micrometastasis. *Clinical Cancer Research*.

[48] Ghossein RA, Bhattacharya S (2000). Molecular detection and characterisation of circulating tumour cells and micrometastases in solid tumours. *European Journal of Cancer*.

[49] Nakanishi H, Kodera Y, Tatematsu M (2004). Molecular method to quantitatively detect micrometastases and its clinical significance in gastrointestinal malignancies. *Advances in Clinical Chemistry*.

[50] Mori M, Mimori K, Ueo H (1996). Molecular detection of circulating solid carcinoma cells in the peripheral blood: the concept of early systemic disease. *International Journal of Cancer*.

[51] Zieglschmid V, Hollmann C, Böcher O (2005). Detection of disseminated tumor cells in peripheral blood. *Critical Reviews in Clinical Laboratory Sciences*.

[52] Ruud P, Fodstad O, Hovig E (1999). Identification of a novel cytokeratin 19 pseudogene that may interfere with reverse transcriptase-polymerase chain reaction assays used to detect micrometastatic tumor cells. *International Journal of Cancer*.

[53] Mitropapas G, Nezos A, Halapas A (2006). Molecular detection of tyrosinase transcripts in peripheral blood from patients with malignant melanoma: correlation of PCR sensitivity threshold with clinical and pathologic disease characteristics. *Clinical Chemistry and Laboratory Medicine*.

[54] Aerts J, Wynendaele W, Paridaens R (2001). A real-time quantitative reverse transcriptase polymerase chain reaction (RT-PCR) to detect breast carcinoma cells in peripheral blood. *Annals of Oncology*.

[55] Stathopoulou A, Ntoulia M, Perraki M (2006). A highly specific real-time RT-PCR method for the quantitative determination of CK-19 mRNA positive cells in peripheral blood of patients with operable breast cancer. *International Journal of Cancer*.

[56] Nagrath S, Sequist LV, Maheswaran S (2007). Isolation of rare circulating tumour cells in cancer patients by microchip technology. *Nature*.

[57] Lurje G, Nagashima F, Zhang W (2008). Polymorphisms in *cyclooxygenase-2* and *epidermal growth factor receptor* are associated with progression-free survival independent of K-ras in metastatic colorectal cancer patients treated with single-agent cetuximab. *Clinical Cancer Research*.

[58] Yen L-C, Yeh Y-S, Chen C-W (2009). Detection of *KRAS* oncogene in peripheral blood as a predictor of the response to cetuximab plus chemotherapy in patients with metastatic colorectal cancer. *Clinical Cancer Research*.

[59] Ito H, Kanda T, Nishimaki T, Sato H, Nakagawa S, Hatakeyama K (2004). Detection and quantification of circulating tumor cells in patients with esophageal cancer by real-time polymerase chain reaction. *Journal of Experimental and Clinical Cancer Research*.

[60] Hoffmann AC, Vallbohmer D, Brabender J (2007). Survivin mRNA levels in peripheral blood from patients with esophageal cancer decrease significantly following surgical resection and are influenced by neoadjuvant chemoradiation. *Digestive Disease Week*.

[61] Parkin DM, Bray F, Ferlay J, Pisani P (2005). Global cancer statistics. *CA: A Cancer Journal for Clinicians*.

[62] Cunningham D, Allum WH, Stenning SP (2006). Perioperative chemotherapy versus surgery alone for resectable gastroesophageal cancer. *The New England Journal of Medicine*.

[63] Miyazono F, Natsugoe S, Takao S (2001). Surgical maneuvers enhance molecular detection of circulating tumor cells during gastric cancer surgery. *Annals of Surgery*.

[64] Ikeguchi M, Kaibara N (2005). Detection of circulating cancer cells after a gastrectomy for gastric cancer. *Surgery Today*.

[65] Hiraiwa K, Takeuchi H, Hasegawa H (2008). Clinical significance of circulating tumor cells in blood from patients with gastrointestinal cancers. *Annals of Surgical Oncology*.

[66] Cohen SJ, Alpaugh RK, Gross S (2006). Isolation and characterization of circulating tumor cells in patients with metastatic colorectal cancer. *Clinical Colorectal Cancer*.

[67] Cohen SJ, Punt CJA, Iannotti N (2009). Prognostic significance of circulating tumor cells in patients with metastatic colorectal cancer. *Annals of Oncology*.

[68] Locker GY, Hamilton S, Harris J (2006). ASCO 2006 update of recommendations for the use of tumor markers in gastrointestinal cancer. *Journal of Clinical Oncology*.

[69] Lurje G, Manegold PC, Ning Y, Pohl A, Zhang W, Lenz H-J (2009). Thymidylate synthase gene variations: predictive and prognostic markers. *Molecular Cancer Therapeutics*.

[70] Lurje G, Zhang W, Yang D (2008). Thymidylate synthase haplotype is associated with tumor recurrence in stage II and stage III colon cancer. *Pharmacogenetics and Genomics*.

[71] Brabender J, Vallböhmer D, Grimminger P (2008). ERCC1 RNA expression in peripheral blood predicts minor histopathological response to neoadjuvant radio-chemotherapy in patients with locally advanced cancer of the esophagus. *Journal of Gastrointestinal Surgery*.

